# The link between gestational diabetes and cardiovascular diseases: potential role of extracellular vesicles

**DOI:** 10.1186/s12933-022-01597-3

**Published:** 2022-09-03

**Authors:** Valeska Ormazabal, Soumyalekshmi Nair, Flavio Carrión, H. David Mcintyre, Carlos Salomon

**Affiliations:** 1 Exosome Biology Laboratory, Centre for Clinical Diagnostics, UQ Centre for Clinical Research, Royal Brisbane and Women’s Hospital, Faculty of Medicine + Biomedical Sciences, The University of Queensland, Building 71/918, Herston, QLD 4029 Australia; 2 Faculty of Biological Sciences, Pharmacology Department, University of Concepcion, Concepción, Chile; 3 Departamento de Investigación, Postgrado y Educación Continua (DIPEC), Facultad de Ciencias de la Salud, Universidad del Alba, Santiago, Chile; 4 Mater Research, Faculty of Medicine, University of Queensland, Mater Health, South Brisbane, Australia

**Keywords:** Cardiovascular disease, Gestational diabetes, Placenta, Extracellular vesicles, Cell communication

## Abstract

Extracellular vesicles are critical mediators of cell communication. They encapsulate a variety of molecular cargo such as proteins, lipids, and nucleic acids including miRNAs, lncRNAs, circular RNAs, and mRNAs, and through transfer of these molecular signals can alter the metabolic phenotype in recipient cells. Emerging studies show the important role of extracellular vesicle signaling in the development and progression of cardiovascular diseases and associated risk factors such as type 2 diabetes and obesity. Gestational diabetes mellitus (GDM) is hyperglycemia that develops during pregnancy and increases the future risk of developing obesity, impaired glucose metabolism, and cardiovascular disease in both the mother and infant. Available evidence shows that changes in maternal metabolism and exposure to the hyperglycemic intrauterine environment can reprogram the fetal genome, leaving metabolic imprints that define life-long health and disease susceptibility. Understanding the factors that contribute to the increased susceptibility to metabolic disorders of children born to GDM mothers is critical for implementation of preventive strategies in GDM. In this review, we discuss the current literature on the fetal programming of cardiovascular diseases in GDM and the impact of extracellular vesicle (EV) signaling in epigenetic programming in cardiovascular disease, to determine the potential link between EV signaling in GDM and the development of cardiovascular disease in infants.

## Introduction

Cardiovascular disease (CVD) is the leading cause of morbidity and mortality worldwide [1]. Risk factors that predispose to CVD include hypertension and multiple metabolic disorders such as obesity, dyslipidemia, and insulin resistance (including type 2 diabetes). The alarming increase in the incidence of type 2 diabetes now predisposes young people to earlier onset of metabolic complications and increased burden of disease [2].

CVD has been considered to be a consequence of adult lifestyle choices (i.e., modifiable risk factors) and genetic predisposition (i.e., non-modifiable risk factors). Attempts to alter lifestyle to reduce risk factors, however, has not mitigated the high rates of CVD [3–5]. Furthermore, genetic polymorphisms associated with diabetes and obesity fail to explain the increase in childhood obesity and type 2 diabetes, both of which are risk factors for CVD [6]. In contrast, there is substantial evidence supporting the involvement of prenatal and postnatal exposure to environmental risk factors in determining life-long disease susceptibility [7–11]. For example, newborns of pregnancies complicated by gestational diabetes mellitus (GDM) are at increased risk of developing type 2 diabetes and CVD in adult life [12]. Despite its high prevalence and association with complications of pregnancy, the underlying pathophysiology of GDM and its effects on fetal metabolism is poorly understood. Current data suggest that the fetus responds to excess maternal nutrition and that GDM affects cellular, molecular, and epigenetic pathways, both in the placenta and in the fetus, predisposing the offspring to subsequent metabolic disease [13–15]. Thus, the effects of a diabetic pregnancy can be considered as an inter-generational vicious cycle, with consequences for the offspring that extend far beyond the neonatal period [16].

Recent studies have identified novel extracellular vesicle (EV) signaling pathways, including exosomal signaling, that mediate maternal–fetal communication. EVs are vesicles released from cells and contain bioactive molecules including proteins and miRNAs that, once released, are capable of regulating proximal and distal cell function [17].The International Society for Extracellular Vesicles (ISEV) endorses the term ‘extracellular vesicles’ (EV) as a generic name for the particles naturally released from cells, delimited by a lipid bilayer, without nucleus, and inability to replicate. However, there are different sub-types of EVs such as EVs of endosome-origin known as “exosomes”, EVs that bud from the plasma membrane known as “ectosomes or microvesicles or microparticles” and EVs released from dying or apoptotic cells known as “apoptotic bodies”. These EV subtypes have overlapping physical and biochemical characteristics and hence assigning an EV to a particular biogenesis pathway based on their physical and biochemical characteristics is difficult [18]. Hence, in this review, we will be using the term small EVs (sEVs) when they are less than 200 nm and medium or large EVs (m/l EVs) when greater than 200 nm.

During pregnancy, EVs have been identified in both the maternal and fetal compartments, and gestational changes in their concentration, contents and bioactivity have been documented [19]. It is now well-established that EV signaling represents a significant pathway in cell-to-cell communication and in regulating cellular functions [20]. Most importantly, EV signaling plays pivotal roles in the pathophysiology of metabolic disorders as key mediators of molecular cross talk between metabolic organs [21–25]. Available data suggest the inevitable implications of EV signaling in the maternal metabolic changes in pregnancy and development of GDM [26–31] Interestingly, the maternal changes in metabolism during pregnancy regulate epigenetic programing during fetal life, leaving a metabolic imprint that defines life-long health and disease susceptibility [32–36]. The focus of this review is to discuss the role of GDM-induced changes in EV signaling that may predispose to postnatal metabolic syndrome and CVD.

## Clinical associations between GDM and cardiovascular diseases in children

Cardiovascular disease is the leading cause of mortality and morbidity worldwide [1] and its effects are significantly compounded in diabetic patients [37]. Although incidence and mortality rates have decreased in some countries, the prevalence of CVD has increased in children and young adults in the last decade [3, 4]. Adults with diabetes have a higher risk of developing CVD than those without diabetes, and the risk of CVD increases with fasting glucose concentrations, even before reaching diagnostic threshold concentrations [38]. The International Diabetes Federation (IDF) estimates that there are 425 million people in the world with diabetes and this number is expected to reach 642 million in the year 2040. Significantly, 91% of all cases are type 2 diabetes [39]. The increased incidence of type 2 diabetes has been attributed to interacting genetic, environmental, and metabolic risk factors. Consistent with this proposal, maternal conditioning before and during pregnancy, including the onset of GDM [40], predisposes to subsequent maternal type 2 diabetes and CVD [41, 42] and also to diabetes and CVD in the offspring [43–46]. A better understanding of this association is crucial to reducing the risk of CVD among children and young adults.

Gestational diabetes mellitus is one of the most common complications of pregnancy and has been defined as glucose intolerance that begins or is first diagnosed during pregnancy [47, 48]. The estimated global prevalence of GDM is between 1 to 14%, depending on the diagnostic criteria, and the population studied [49]. Recent studies identified an increase in the prevalence of GDM that correlates with an increase in urbanization, reduction in physical activity, excess weight gain during pregnancy, and exposure to obesogenic environments [50–52].

Alterations in the metabolism of lipids and carbohydrates are common conditions experienced by women with GDM. Several studies report a high cardio-metabolic risk in children exposed to GDM in the womb, considering it an independent risk factor for glucose intolerance and CVD [53, 54]. Prenatal exposure to maternal diabetes is associated with congenital heart disease, obesity and diabetes in the offspring [55, 56]. Exposure to altered metabolic states at critical periods during fetal development, thus, may have long-term impact, generating a metabolic memory that increases the risk of CVD in adulthood [43, 57–60].

Consistent with the hypothesis that GDM predisposes to CVD in the offspring, biomarkers of CVD risk (including blood lipid profile, obesity or overweight, alterations in blood pressure, and insulin resistance) are evident in children born of mothers with GDM [12, 42, 44, 61–64]. Furthermore, children (3 years and older) born of pregnancies complicated by GDM have increased systolic and diastolic blood pressure [12, 53], low plasma high-density lipoprotein (HDL) concentrations, hypertriglyceridemia [65], glucose intolerance [63], or insulin resistance [53, 63, 66], as well as increased adiposity [12, 63, 67, 68].

In the adolescent offspring of pregnancies complicated by GDM, abnormal blood pressure, lipid profiles [69] and glucose tolerance [70, 71], high cholesterol concentrations (in females) and increased systolic blood pressure (in males), and a predisposition to type 2 diabetes or pre-diabetes [72] have been reported. The higher risk of developing metabolic syndrome in offspring born to GDM mothers could be attributed to the increase in fetal adiposity or birth weight as a consequence of GDM [73]. As obesity is a major risk factor that contributes directly to dyslipidemia, type 2 diabetes, hypertension and CVD [74], increased birth weight and adiposity can be contributing factors to the development of childhood and early adulthood metabolic disorders. Hyperinsulinemia in-utero is associated with a 17-fold increase in incidence of metabolic syndrome and a tenfold increase in overweight, independent of birth weight [62]. Overweight is observed in children of 4 to 5 years of age, born to mothers with GDM and is associated with increased birth weight, maternal obesity [75], and/or altered lifestyle during childhood [76]. Children with a high birth weight who are exposed to an intrauterine environment of diabetes or maternal obesity have an increased risk of developing metabolic syndrome [56, 77].

Further, pregnancies complicated with GDM and maternal obesity pose a higher risk for the development of metabolic syndrome in offspring, including CVD [69, 76, 78–80]. Maternal pre-gestational [66, 81, 82] and gestational body weight are strong predictors of body mass index (BMI) and percentage total body fat [67] in female but not male offspring [42]. Prenatal exposure to GDM concomitant with maternal obesity or overweight is a high-risk factor for high BMI in offspring [79, 83]. However, for the development of offspring overweight and obesity, the risk attributed from maternal obesity is higher compared to GDM [76, 80]. In addition, in a larger study, of 2,432,000 children over a 40-year period, the offspring of GDM pregnancies had a 29% increased rate of early-onset CVD [78] that was further increased in association with comorbidities such as maternal obesity [78]. Table 1 summarizes the clinical consequences of gestational diabetes in the offspring and its impact on CVD development.

**Table 1:** Clinical consequences of gestational diabetes in offspring

Cohort	Clinical findings	References
164 Chinese children at a median age of 8 years (range: 7–10 years)	Maternal GDM increases the offspring’s cardiometabolic risk	[53]
Eighty-nine children (mean age 9.1 years, 93% Caucasian)	School-age children of mothers with GDM are at risk of IGT and being overweight	[63]
Studied 1,238 mother–child	Children exposed to GDM have higher adiposity, which may mediate higher systolic blood pressure in these children	[12]
Sixty-eight children	Among these children, 45 (66%), 17 (25%), 5 (7%), and 1 (1.5%) had zero, one, two, or three metabolic markers of IR, respectively	[65]
Case mothers who had GDM/GIGT in pregnancy (cases; n = 90) and normoglycaemic control women (n = 99) and their daughters underwent lifestyle assessment and metabolic tests 15-years post-partum	Case daughters have increased risk of central adiposity and insulin resistance, whereas maternal obesity strongly predicted daughters’ BMI percentile and per cent of body fat	[67]
One hundred and twenty-nine adolescents who were assessed for their cardiometabolic risks at 8 years of age were reassessed at 15 years of age	Adolescent offspring of mothers with GDM had similar blood pressure, plasma lipid profile, and a rate of abnormal glucose tolerance as control subjects. In-utero hyperinsulinemia was associated with a 17-fold increase in metabolic syndrome and a tenfold increase in overweight at adolescence, independent of birth weight, Tanner stage, maternal GDM status, and mother’s BMI	[62]
A total of 970 mothers who had joined the Hyperglycemia and Adverse Pregnancy Outcome study were reevaluated, together with their child born during the study period, 7 years after delivery	Maternal hyperglycemia in pregnancy is independently associated with offspring’s’ risk of abnormal glucose tolerance, obesity, and higher BP at 7 years of age. Its effect on childhood adiposity was apparent only in girls, not boys	[42]
BMI measurements were collected at age 2, 8, and 11 years from 232 offspring of mothers with GDM (OGDM) and compared with those from 757 offspring of mothers with type 1 diabetes (OT1D) and 431 offspring of nondiabetic mothers (ONDM)	Overweight and insulin resistance in children is increased in OGDM compared with OT1D or ONDM. The finding that overweight risk is associated mainly with maternal obesity suggests that familial predisposition contributes to childhood growth in these offspring	[66]
Studied height and BMI standard deviation score (SDS) of the OGDM group, up to the age of 14 years, with subgroup analysis comparing Large for Gestational Age (LGA) with non-LGA at birth as a reflection of the intrauterine environment	Until early adolescence, OGDM had a BMI that is 0.5 SDS higher than that of the Dutch background population. LGA OGDM appear to be at particularly higher risk of being overweight during adolescence compared with non-LGA OGDM, putting them also at a higher lifetime risk of being overweight and developing obesity. Offspring of mothers with type 2 diabetes (ODM2) showed the highest BMI SDS values and had an average BMI SDS of + 1.6 until the age of 14, when it became + 2 SD	[82]
Prevalence of overweight and abdominal obesity at age 16 years and odds ratios (ORs) for prenatal exposures to maternal prepregnancy overweight and GDM. Study prospective longitudinal Northern Finland Birth Cohort of 1986 (N = 4,168)	Maternal pre-pregnancy overweight is an independent risk factor for offspring overweight and abdominal obesity at age 16 years. The risks are highest in offspring with concomitant prenatal exposure to maternal pre-pregnancy overweight and GDM	[80]
Studied 255 obese adolescents with normal glucose tolerance. All of them were investigated for in utero exposure to GDM	Obese youth exposed in-utero to GDM show early inability of the beta cell to compensate adequately in response to decreasing levels of insulin sensitivity	[70]
HAPO Follow-up Study (FUS) included 4,160 children ages 10–14 years	Offspring exposed to untreated GDM in-utero are insulin resistant with limited β-cell compensation compared with offspring of mothers without GDM. GDM is significantly and independently associated with childhood IGT	[71]
(HAPO) Study evaluated the long-term outcomes (4697 mothers and 4832 children	Among children of mothers with GDM vs those without it, the difference in childhood overweight or obesity defined by body mass index cutoffs was not statistically significant; however, additional measures of childhood adiposity may be relevant in interpreting the study findings	[61]
Data from 7355 mother–child dyads of the German Perinatal Prevention of Obesity cohort	The postulated increased risk of overweight and abdominal adiposity in offspring of mothers with gestational diabetes cannot be explained by maternal BMI alone and may be stronger for childhood obesity than for overweight	[200]
At a mean age of 24.1 ± 1.3 years, were classified offspring as offspring of mothers with GDM regardless of the prepregnancy BMI (OGDM; n = 193); normoglycemic mothers with prepregnancy overweight/obesity (ONO; n = 157); and normoglycemic mothers with prepregnancy BMI < 25 kg/m^2^ (controls; n = 556)	Adult offspring of mothers with GDM have increased markers of insulin resistance and a more atherogenic lipid profile	[69]
Prospective cohort study included 10,412 mother–child pairs tested for GDM with IADPSG criteria	The associations between GDM diagnosed using IADPSG criteria and BMI Z-score and the risk for overweight/obesity in offspring were largely explained by maternal pre-pregnancy BMI at the age of 1–4 years	[75]
Study in 1967 mother–child pairs	Offspring of mothers with both GDM and HDP had a higher BMI than children born from a normotensive and normoglycemic pregnancy Maternal GDM alone or joint GDM and HDP were associated with increased ratios of offsprings being overweight	[68]
A total of 298 offspring (202 offspring of GDM mothers and 96 offspring of mothers with impaired glucose tolerance [IGT]) participated in the study	In offspring of GDM mothers, CVD risk factors were positively correlated with age, except for lipid profiles	[201]
It was examined associations of maternal GDM (n = 92 cases out of 597) with mean serum lipid levels in the offspring	GDM exposure was associated with higher total- and low-density lipoproteins (LDL)- cholesterol in girls. In boys, maternal GDM corresponded with higher SBP (systolic blood pressure). Maternal GDM is related to offspring lipid profile and SBP in a sex-specific manner	[202]
Follow-up study of 1066 primarily Caucasian women aged 18–27 yr in the Center for Pregnant Women with Diabetes, Rigshospitalet, Copenhagen, Denmark	The risk of overweight was doubled in offspring of women with diet-treated GDM or type 1 diabetes, whereas the risk of metabolic syndrome was 4- and 2.5-fold increased, respectively. Offspring risk of metabolic syndrome increased significantly with increasing maternal fasting blood glucose as well as 2-h blood glucose (during oral glucose tolerance test)	[72]
Follow-up study of 567 offspring, aged 18–27 years	Fasting plasma levels of glucagon-like peptide-1 (GLP-1) were lower in the two diabetes-exposed groups compared to offspring from the background population. Increasing maternal blood glucose during oral glucose tolerance test (OGTT) in pregnancy was associated with reduced postprandial suppression of glucagon in the offspring. Lower levels of GLP-1 and higher levels of glucagon during the OGTT were present in offspring characterized by overweight or prediabetes/type 2 diabetes at follow-up, irrespective of exposure status	[203]

Maternal hyperglycemia results in increased placental transfer of glucose and amino acids to the fetus leading to fetal hyperinsulinemia. This fetal insulinemia results in short-term consequences such as fetal overgrowth, adiposity, and hypoglycaemia [84].This can also induce changes in fetal cardiovascular function characterized by elevated concentrations of endothelial dysfunction markers (E-selectin and VCAM1), and leptin [44], increased thickness of the aortic intima-media, and arterial stiffness [85].The available data support the hypothesis that in-utero exposure to hyperinsulinemia and hyperglycemia has lasting effects on gene expression and known risk factors for CVD in the offspring [85]. Exposures to adverse metabolic environments during early life, thus, leave an imprint that determines the long-term health of an individual [86]. Compelling data implicates epigenetic mechanisms in regulating these effects and their subsequent transmission to future generations [87, 88].

## Fetal programming for the development of cardiovascular disease

Until recently, it was widely accepted that the development of chronic metabolic disease is a consequence of inherited genetics and a lifetime of exposure to suboptimal lifestyle factors (e.g., inactivity, diet). These factors, however, only explain 10% of the origin of chronic non-communicable diseases [89]. The polymorphisms associated with diabetes and obesity, similarly, fail to explain the notable increase in childhood obesity and type 2 diabetes, which are risk factors for CVD [6]. Additionally, children born after maternal onset of diabetes may have a higher risk of obesity and type 2 diabetes when compared to their siblings born before maternal disease onset [90]. This observation is significant in that it identifies maternal diabetes as an independent contributing factor for obesity and type 2 diabetes in the offspring. Furthermore, it is consistent with the tenet of developmental origins of the health and diseases (DOHaD) [43] and the role of epigenetics in fetal adaptation to maternal metabolic status [91].

Epigenetic regulation is defined as inheritable changes in gene expression in response to environmental stimuli that do not include changes in nucleotide sequence [92–94]. Epigenetic modifications include: DNA methylation on CpG islands [95, 96], post-translational modifications of histone proteins, and regulation of expression by non-coding RNA (such as, miRNA) [97, 98]. Changes in epigenetic patterns can result in impaired gene expression, which itself can have many downstream effects, including changes in disease risk, stress response, and changes in cellular metabolism [99–101].

As alluded to above, GDM predisposes offspring to chronic diseases. One mechanism by which this may occur is fetal metabolic reprogramming, involving epigenetic changes [102]. Differential DNA methylation profiles have been identified in umbilical cord blood and /or placenta obtained from infants of GDM pregnancies compared to normoglycemic pregnancies [15, 34, 36, 103–105]. Differentially methylated genes include genes associated with CVD, metabolic diseases specifically type 2 diabetes, and immunological and endocrine disorders [51, 103].

For example, leptin, an adipokine involved in energy balance and appetite, is differentially methylated in placental tissues obtained from pregnancies complicated by GDM [106] and is dependent on glucose concentrations [107]. Leptin methylation is associated with decreased leptin mRNA abundance in placental tissues [108] and decreased leptin concentrations in maternal serum and umbilical cord blood, in the third trimester. Leptin concentrations are negatively correlated with glucose concentrations at 2 h post-OGTT. These data highlight an in-utero effector pathway whereby GDM may induce long-lasting epigenetic modification of metabolism that increases the likelihood of obesity in later life. Similarly, GDM-responsive pathways have been reported for other metabolic pathway genes including MEST (human mesoderm-specific transcript, a gene implicated in fetal and placental growth) and ABCA1 (a member of the ATP-binding cassette family).

MEST is an imprinted gene implicated in placental development. During the first trimester, the translational product of MEST, mesoderm-specific transcript homolog protein, is highly expressed in extra-villous trophoblast cells [109]. Aberrant methylation of MEST is associated with dysregulation of placentation and complications of pregnancy [109]. Placental tissue and cord blood obtained from pregnancies complicated by GDM display significantly decreased methylation of MEST when compared to tissues obtained from normoglycemic pregnancies [32]. Decreased methylation of MEST is also characteristic of obese adults. In mice, the loss of MEST differentially methylated domains is strongly associated with the accumulation of triacylglycerol within the placenta and abnormal mitochondrial function [110]. In support of the role of MEST in lipid metabolism, overexpression of MEST in transgenic mice increases the size of adipocytes [111], while global knockdown of MEST reduces adiposity [112]. In the setting of GDM, decreased methylation of MEST may alter placental function and lipid metabolism, having a long-lasting effect on the epigenome of offspring and contributing to a predisposition to obesity throughout life [32, 113].

**Fig. 1:**
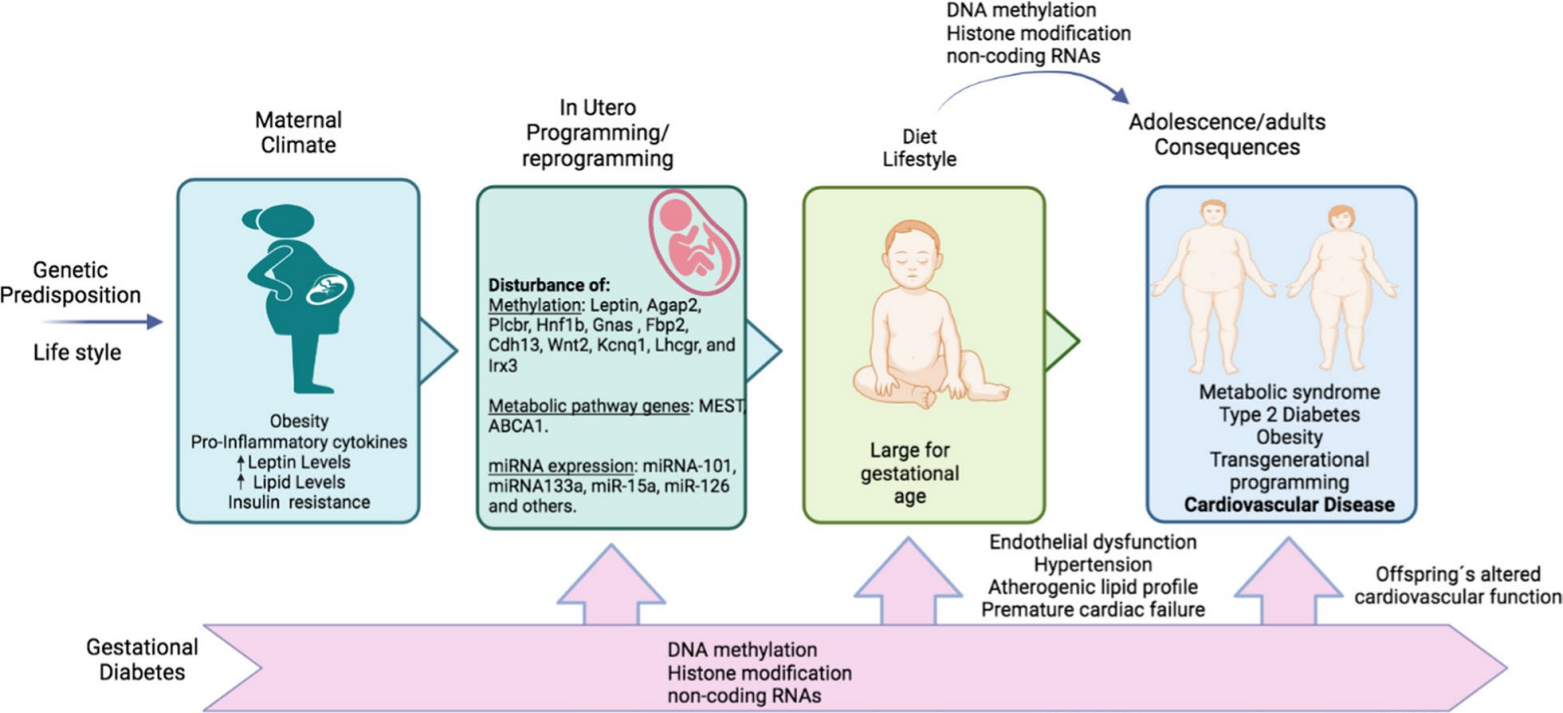
Offspring consequences due to maternal GDM, and exposure to environmental factors throughout its lifetime, leading to alterations in epigenetic programming

DNA methylation in the ABCA1 transporter gene has previously been associated with CVD. ABCA1 is a transporter that transfers cholesterol from cells to apolipoproteins A-1 (apo-A1), which contributes to the formation of nascent HDL [114]. In a study by Houde and collaborators, the association between the maternal metabolic profile and the methylation levels of the ABCA1 transporter gene, in placenta and umbilical cord blood in pregnancies with GDM was evaluated [35]. Results indicated that the methylation levels of the ABCA1 gene on the maternal side of the placenta correlated with the maternal HDL cholesterol levels and glucose levels observed at 2 h on the diagnostic OGTT. On the fetal side of the placenta, methylation levels of the ABCA1 gene were associated with triglyceride levels in umbilical cord blood. Variability of DNA methylation on both sides of the placenta was also associated with ABCA1 mRNA levels. In contrast, cord blood ABCA1 gene methylation levels were negatively correlated with maternal glucose levels 2 h after OGTT [35]. These results suggest that epigenetic adaptations to the uterine environment may contribute to triggering long-term susceptibility to dyslipidemia and developing CVD (Fig. 1).

In animal models, pancreas from offspring of mothers with GDM showed changes in DNA methylation patterns of several genes involved in signaling pathways associated with glucose and lipid metabolism, such as, Agap2, Plcbr, Hnf1b, Gnas, Fbp2, Cdh13, Wnt2, Kcnq1, Lhcgr, and Irx3 [115]. This suggests that changes in the DNA methylation profile in the pancreas genome are associated with metabolic alterations and increased susceptibility to developing type 2 diabetes, obesity, and cardiovascular risk in adult life [115].

DNA methylation is not the sole epigenetic mechanism involved in GDM. More recently, miRNAs have also been investigated as possible mediators of epigenetic modifications in GDM. During GDM, maternal hyperglycemia can favor the transfer of glucose from the mother to the fetus through the fetal placental circulation, inducing endothelial dysfunction in the fetal micro- and macro-circulation, which is associated with an increase in the susceptibility of developing type 2 diabetes along with CVD [116]. Exposure to high glucose levels has been proposed to induce epigenetic changes that negatively affect endothelial function. It has been demonstrated in tests carried out on human umbilical vein endothelial cells (HUVEC) cells, obtained from pregnancies with GDM, that both high glucose and GDM induce endothelial dysfunction through a mechanism mediated by an increase in the expression of miRNA-101 and a reduced expression of histone methyl transferase enhancer of zester homolog-2 (EZH2-ß), which reduces trimethylation of lysine 27 in Histone 3 [117]. These results also showed that the inhibition of miRNA-101 reestablishes cell function and increases the expression of EZH2-ß along with favoring cell survival. Therefore, an altered miRNA-101/EZH2-ß ratio could contribute to the endothelial dysfunction observed in GDM.

In addition, a key event in cardiac myogenesis has been determined to occur during pregnancy, in which fetal exposure to metabolic diseases may dramatically alter postnatal muscle development and metabolism [118, 119]. miRNA133a has been shown to participate in muscle differentiation and regulate the mitochondrial function of muscle cells. Alterations in miRNA133a expression were associated with severe myopathies and mitochondrial dysfunction [120]. In addition, murine models exposed to GDM during fetal development showed muscle lipotoxicity, which was evidenced by diacylglycerol accumulation, insulin resistance, reduced miR-133a expression, and elevated Nix expression [121], a known mediator of mitophagy and programmed cell death [122].

Members of the miR-15 family can alter the expression or function of distinct proteins involved in the insulin signaling pathway, thus affecting insulin sensitivity and secretion. Coincidentally, skeletal muscle biopsies of people between 26 and 35 years of age whose mothers were previously diagnosed with GDM have shown increased expression in skeletal muscle of miR-15a and miR-15b. Additionally, miR-15a expression was positively associated with fasting plasma glucose, 2 h plasma glucose and HbA1c levels, which may contribute to the development of metabolic diseases and high cardiovascular risk [123].

miRNAs have been implicated in the epigenetic regulation of key metabolic and inflammatory pathways and their expression is modified in the face of stress situations or metabolic adaptations [124]. The majority of cells selectively secrete miRNAs. In addition, changes in the profile of miRNAs secreted into the blood have been associated with several pathologies, where they have been considered as potential biomarkers of health or disease status [125, 126]. There are numerous publications in which changes in the circulating miRNA profile have been observed in pre-diabetic and diabetic patients, such as an increase in the levels of miR-192 and miR-193b, and a decrease in miR-126, and miRNA15a, among others [127–129].

A recent meta-analysis has found that 40 circulating miRNAs, including miR-21, miR-29a, miR-34a, miR-103, miR-107, miR-126, miR-132, miR-142-3p, miR-144 and miR-375, are significantly dysregulated in type 2 diabetes [130]. Another study demonstrates a decrease in miRNA levels; miR-20b, miR-21, miR-24, miR-15a, miR-126, miR-191, miR-197, miR-223, miR-320, and miR-486 in type 2 diabetes, and a modest increase in miR-28-3p [131].

These results strongly suggest the potential utility of miRNAs as diagnostic and follow-up biomarkers for therapeutic interventions in type 2I diabetes [132, 133]. Since a single miRNA can target multiple genes, and multiple miRNAs share common targets, miRNAs are particularly well suited for analyzing metabolic pathways and processes. In circulation, miRNAs can be found bound to protein complexes [134], HDL [135] or within EVs [136]. miRNA-containing EVs have been shown to participate in tumor progression and chemoresistance [137], as well as in the development of atherosclerosis [138], and diabetes [139], amongst other pathologies [140, 141].

## Extracellular vesicular signaling in epigenetic programming in cardiovascular diseases

The development of diabetes involves alterations in insulin-sensitive tissues. This leads to a state of glucose intolerance or prediabetes, that eventually produces overt diabetes when pancreatic β cells are incapable of coping with an increased demand for insulin [142]. Communication between different tissues is essential for the maintenance of glucose homeostasis [143]. Cells can communicate with neighboring cells or with distant cells specifically through the secretion of EVs [144]. As previously mentioned, the term "EVs" describes a class of small membrane-enclosed vesicles (30–2000 nm) that contain a wide variety of messenger biomolecules, such as lipids, proteins, and nucleic acids, which are a representation of the metabolic and functional state of the cells of origin. Most cell types release these vesicles, which are able to cross biological barriers, and can therefore be found in a variety of fluids, including blood, urine, saliva, breast milk and cerebrospinal fluid, among others [145]. Cells can secrete different types of EVs, which are classified according to their subcellular origin and size [146]. The smallest EVs are known as exosomes (50–150 nm), which are generated within endosomes or multivesicular bodies (MVB) and are secreted when these compartments fuse with the plasma membrane. Other EVs are much larger, such as microvesicles (200–1000 nm) and apoptotic bodies (over 1000 nm), both of which are formed by sprouting from the plasma membrane [147]. EVs express their own surface molecules, which allow them to target specific recipient cells. Once bound to a target cell, EVs can induce signaling through receptor-ligand interaction or can be internalized by endocytosis and/or phagocytosis, or may fuse with the target cell's membrane to deliver its content, thereby modifying the physiological state of the recipient cell [148].

EVs can transfer proteins and nucleic acids (small and long non-coding RNAs) that can mediate epigenetic programming in recipient cells, including DNA methylation, histone modification, and posttranscriptional regulation of RNA [149, 150]. DNA methylation involves addition of methyl groups to the cytosine residues of DNA, converting them to 5-methylcytosine and this is mediated by DNA methyltransferase (DNMT) enzymes. DNA methylation leads to stable long-term gene silencing and demethylation leads to upregulation of gene transcription. EVs can transfer molecular factors that can mediate DNA methylation in target cells [151]. For example, EVs derived from leukemia cells contain breakpoint cluster region–Abelson leukemia gene human homolog 1 (BCR-ABL1) mRNA, which when transferred to normal cells upregulate the methyltransferases and global DNA hypermethylation resulting in malignant transformation [149]. sEVs derived from endothelial cells deliver miRNAs (miR-21-5p and miR-217) that target the DNA methyl transferase 1 (DNMT1) in recipient cells and suppress genes associated with cell proliferation conferring senescence phenotype in recipient cells [150]. The transfer of senescence signals has a key role in the development of age-related CVD and diabetes.

The posttranscriptional modification of histones, particularly acetylation and methylation can regulate gene transcription [151]. EVs can be involved in the transfer of factors that can mediate these histone modifications and regulate chromatin remodeling. For example, in ischemic heart disease, sEV-mediated transfer of Heat Shock Factor 1 (HSF1) interacts with the promotor region of miR-34 and alters the methylation profile of Histone 3 protein. This downregulates miR-34 expression and rescues the cardiomyocytes from apoptosis via HSF1/miR-34a/Heat shock protein (HSP)70 pathway [152]. Also, brain tumor cells secreting EVs carrying linker Histone H1 can bind DNA and regulate DNA methylation and histone modification and mediate tumorigenesis [153].

Long non-coding RNAs (lncRNA) consist of approximately 200 nucleotides and can regulate nuclear architecture and thereby modify gene transcription [154]. sEV-mediated transfer of lncRNA and their functional effects on gene expression in target cells have been reported in CVDs such as atherosclerosis and myocardial infarction. Given that lipid induced inflammation is a characteristic feature of CVD, cells exposed to LDL secrete sEVs containing lncRNA  Growth arrest specific (GAS)5, and regulate apoptosis in target cells (macrophages and endothelial cells), and contribute to the pathology of atherosclerosis [155]. Further, sEVs containing lncRNA metastasis-associated lung adenocarcinoma transcript 1 (MALAT1) derived from endothelial cells activate nuclear factor erythroid 2-related factor (NRF2) signaling in dendritic cells, increasing their maturation and progression to an atherosclerotic state [156]. Also, MALAT1 containing sEVs from endothelial cells activate M2 macrophage polarization [157] and formation of neutrophil extracellular traps and contribute to the pathology of atherosclerosis [158]. In myocardial infarction, MALAT1 containing sEVs derived from cardiomyocytes attenuated miR-92a expression in target cells and help neovascularization [159]. Also, sEV mediated transfer of lncRNA H19 regulate the expression of miR-675 and activate pro-angiogenic factors leading to cardioprotective effects in myocardial infarction [160]. On the other hand, sEVs derived from hypoxic cardiomyocytes were enriched in ncRNA AK139128 which stimulated apoptosis and exacerbated the myocardial infarction in rat models [161].

LncRNAs are implicated in the pathophysiology of insulin resistant states such as diabetes. For example, transfer of lncRNA-p3134 via sEVs could positively regulate glucose-stimulated insulin secretion by promoting key regulators such as Pdx-1, MafA, GLUT2 and Tcf712, in beta cells and enrichment of this lncRNA in serum EVs is reported in type 2 diabetes compared to normoglycemic subjects [162]. This could be a compensatory mechanism of increasing beta cell function in an insulin resistant state. The protective mechanism of sEV transfer of lncRNA has been reported in diabetic complications as well [163]. In diabetic retinopathy, mesenchymal stem cell-derived EVs transfer lncRNA SNHG7 to retinal microvascular endothelial cells, which negatively regulate the miR-3aa-5p/X-box binding protein (XBP)1 axis. By targeting the miR-3aa-5p/XBP1 axis, sEV lncRNA SNHG7 inhibits high glucose induced endothelial mesenchymal transition and tube formation and impairs the pathogenesis of diabetic retinopathy [163]. Also, sEV transfer of lncRNA H19 from mesenchymal stem cells to fibroblast, target miR-152-3p/PTEN pathway. This negatively regulates the expression of miR-152-3p which in turn upregulates PTEN expression and leads to increased proliferation, and migration, and decreases apoptosis, which in turn improves diabetic wound healing [164]. As previously mentioned, ageing is a risk factor for CVD, and it has been reported that mesenchymal stem cell-derived sEVs prevent ageing induced cardiovascular dysfunction by transfer of lncRNA MALAT1. sEV mediated transfer of MALAT1 inhibits the NF-kB/Tumour necrosis factor (TNF)-α signaling pathway and impairs the ageing process [165]. In addition, circulating levels of sEV MALAT1 is significantly decreased in patients with type 2 diabetes [166].

Another subset of non-coding RNAs that has regulatory roles in gene transcription and protein translation are the circular RNAs, which are covalently closed RNAs and can act as miRNA sponges. Emerging evidence implicates the role of sEV associated circular RNA in CVD, and associated metabolic disorders. The circular RNA circRNA‑0006896 in serum sEVs was implicated in carotid plaque destabilization, a key event in ischemic heart disease. Transfer of sEV associated circRNA‑0006896 in endothelial cells resulted in decreased expression of miR-1264 and increased levels of DNMT1, leading to endothelial cell proliferation and migration and carotid plaque destabilization [167]. High glucose stimulated endothelial cells secrete sEVs containing circular RNA circRNA-0077930 which when delivered to vascular smooth muscle cells cause vascular senescence [168]. sEV circular RNA circ_DLGAP4 promote cell proliferation and fibrosis in diabetic kidney disease and exacerbates this diabetic complication by sponging miR-143 and targeting the ERBB3/NF-kB/Matrix metalloproteinase (MMP)-2 axis [169].

On the other hand miRNAs which are 19–24 nucleotides long can regulate gene expression at the post-transcriptional level by RNA-induced gene silencing and this can be considered an epigenetic mechanism under a broader definition of epigenetics [170]. The role of EVs in the transfer of miRNAs is well-established [171, 172], and EV-miRNAs play critical roles in the development and pathophysiology of CVD, including a contribution to the development of risk factors associated with CVD such as diabetes and obesity. Diabetic heart disease is characterized by injury to cardiac microvascular endothelial cells and cardiomyocyte dysfunction. During the initial course of diabetes, hyperglycemia can lead to endothelial dysfunction that contributes to the development of serious vascular complications [173]. There are reports which suggest that despite achieving good glycemic control, vascular damage persists due to induced changes in gene expression, a phenomenon called metabolic memory, which affects different cell types [174]. Currently, a series of studies have shown that miRNA containing EVs can regulate gene expression and modulate endothelial function, inflammation and cellular senescence, key phenomena in cardiovascular function [175]. miR-126 is the most widely studied miRNA in type 2 diabetes and reported to be associated with the maintenance of vascular function [176] and endothelial function [131]. High glucose levels have been shown to reduce miR-126 levels in the apoptotic bodies of endothelial cells. Likewise, the content of miR-126 is reduced in circulating vesicles in diabetic patients [131]. This finding is crucial, since miR-126 has been shown to play an important role in vascular endothelial repair [177]. A report using a cellular endothelial model demonstrated that hyperglycemia weakens potentially protective intercellular communication mechanisms by affecting the content of miRNA-126-3p and the function of endothelial microparticles [177]. Supporting these findings, EVs isolated from Goto-Kakizaki (GK) diabetic rat cardiomyocytes contained higher levels of miR-320 and lower levels of miR-126 compared to EVs obtained from healthy controls [178]. In particular, EVs which contained miRNA-320 lead to a decrease in migration, and proliferation capabilities, along with diminished angiogenesis in adjacent cardiac endothelial cells. Such events may happen through the negative interaction of these EVs with the recipient cells target genes, such as IGF-1, Hsp20 and Ets2, which damage the angiogenic function of these cells [178]. Overexpression of Hsp20 in diabetic mice significantly attenuates cardiac dysfunction and cardiovascular hypertrophy [179]. Interestingly, cardiomyocyte-derived sEVs obtained from mice which overexpress Hsp20, have high levels of p-Akt, Hsp20, survivin, and superoxide dismutase (SOD)1, and have a protective effect in vitro against cell death triggered by hyperglycemia [179].

García and collaborators demonstrated that under glucose starvation conditions, H9C2 cardiomyocytes increase sEV secretion and that their miRNA and protein cargo is dependent on glucose concentrations [180]. Under starvation conditions, EVs are enriched in glucose transporters and enzymes involved in glucose metabolism, which leads to an increased rate of glucose uptake and glycolysis in cardiac endothelial cells [180]. Additionally, another report describes that sEVs derived from cardiomyocytes alter the function of endothelial cells and stimulate angiogenesis, which is dependent on glucose concentration [181].

Furthermore, high glucose levels lead to an increase in the expression of mammalian sterile 20-like kinase 1 (Mst1) kinase in sEVs from diabetic cardiac endothelial cells and cardiomyocytes. In addition, an increase in Mst1 expression has been associated with diabetic heart disease due to its role as regulator of autophagy and apoptosis [182]. Interestingly, cardiomyocytes exposed to sEVs obtained from diabetic cardiac endothelial cells show reduced glucose uptake since Mst1 interrupts the translocation of GLUT4 to the membrane, thus affecting cardiomyocyte metabolism [182]. Further, several reports have shown that Mst1 contributes to the development of diabetic cardiomyopathy by inhibiting the expression of sirtuin 3 (Sirt3) [183], which plays a crucial role in mitochondrial homeostasis and confers a protective role against the onset and development of diabetic cardiomyopathy [184]. On the other hand, obesity is frequently associated with metabolic disease and cardiovascular risk. In an animal model, obesity has been shown to increase the levels of miR-122, miR-192, miR-27a-3p and miR-27b-3p when compared to the miRNA profile of plasma sEVs derived from lean animals. Interestingly, treatment of lean mice with sEVs isolated from obese mice induces glucose intolerance and insulin resistance. Furthermore, the administration of control sEVs transfected with obesity-associated miRNA mimics strongly induces glucose intolerance in lean mice and produces central obesity and hepatic steatosis [185], thus contributing to cardiovascular damage. Table 2 summarizes the key functional studies on EV signaling in CVD, and associated risk factors.

**Table 2:** Epigenetic programming induced by EVs on CVDs development

Epigenetic modification	Metabolic disorder	Findings	References
DNA methylation			
	Senescence induced vascular effects	Endothelial cells secrete sEVs enriched in miRNAs miR-21 and miR-217 which target DNMT1 and SIRT1, affecting DMA methylation, cell replication, and spread ageing signals in cells	[150]
Histone modification			
	Ischemic heart disease	Exosomal transfer of HSF1 leads to histone methylation and chromatin remodeling at the promoter region of miR-34, and this could rescue cardiomyocyte apoptosis in ischemic heart disease	[152]
Long non-coding RNAs			
	Atherosclerosis	lncRNA GAS5 is transferred via exosomes and regulates apoptosis in macrophages and endothelial cells aggravating the atherosclerotic condition	[155]
	Atherosclerosis	sEVs derived from endothelial cells contain MALAT1 and when transferred to dendritic cells, accelerate dendritic cell maturation and progression of atherosclerosis by interacting with NRF2	[156]
	Atherosclerosis	MALAT1 in sEVs derived from endothelial cells promote M2 macrophage polarization and contribute to atherosclerosis progression	[157]
	Atherosclerosis	Low density lipid treated endothelial cells secrete sEVs with MALAT1 and it triggers hyperlipidemia, the inflammatory response, and neutrophil extracellular traps that accelerate the pathology of atherosclerosis	[158]
	Myocardial infarction	sEV transfer of MALAT1 between hyperbaric oxygen treated cardiomyocytes leads to miR-92a suppression and neovascularization	[159]
	Myocardial infarction	Transfer of lncRNA H19 via sEVs between cardiomyocytes reduce apoptosis and perform a cardioprotective function	[160]
	Myocardial infarction	Transfer of lncRNA AK139128 via sEVs in the presence of hypoxia leads to cardiomyocyte apoptosis, exacerbating the pathology of myocardial infarction	[161]
	Type 2 diabetes	Exosomal transfer of lncRNA-p3134 positively regulates glucose-induced insulin secretion by promoting key regulators such as Pdx-1, MafA, GLUT2, and Tcf712 in beta cells. This lncRNA-p3134 is upregulated in serum exosomes in type 2 diabetic patients indicating a compensatory mechanism	[162]
	Diabetic retinopathy	Mesenchymal stem cells secrete exosomes containing lncRNA SNHG7 which when transferred to retinal microvascular endothelial cells impairs the miR-34a-5p/XBP1 pathway and protects from diabetic retinopathy pathogenesis	[163]
	Ageing	Mesenchymal stem cells secrete exosomes containing lncRNA MALAT1 which inhibits the NF-kB/TNF-α signaling pathway and impairs the aging process	
	Diabetic wound healing	Mesenchymal stem cells secrete exosomes containing lncRNA H19 which when transferred to fibroblast increase fibroblast proliferation and impair apoptosis by upregulating PTEN by targeting miR-152-3p	[164]
Circular RNA			
	Atherosclerosis	Transfer of circRNA‑0006896 via sEVs target the miR1264‑DNMT1 pathway in target cells and mediate endothelial cell proliferation and migration	[167]
	Ageing	CircRNA-0077930 containing sEVs secreted by endothelial cells downregulate miR-622 and upregulate KRAS, p21, p53, and p16 expression and regulate vascular senescence	[168]
miRNAs			
	Cardiomyopathy	sEV mediated transfer of miR-320 to endothelial cells provide an anti-angiogenic function in animal models, and leads to diabetes mellitus-induced myocardial vascular deficiency	[178]
	Hypertension	sEV mediated transfer of miR-155-5p to fibroblasts enhances the expression of angiotensin II and angiotensin converting enzyme, and promotes vascular remodeling	[199]
	Atherosclerosis	sEVs containing miR-223 derived from thrombin activated platelets inhibit TNF-α stimulated endothelial cell inflammation and play a protective role in atherosclerosis	[204]
	Myocardial infarction	Mesenchymal stem cell derived sEVs containing miR-22 target methyl CpG binding protein and reduce cardiac fibrosis	[205]
	Atrial fibrillation	Myofibroblast derived sEVs containing miR-21-3p upregulate L-type calcium channel CaV1.2 in target cells and contribute to atrial fibrillation	[206]
	Atherosclerosis	Mesenchymal stem cell derived sEVs containing miR-let7 mediate macrophage migration and M2 polarization via the IGF2BP1 and HMGA2 pathways, and ameliorate atherosclerosis	[207]

## Potential role of EVs in epigenetic programming in GDM

Maternal glucose is the primary nutrient that sustains fetal growth and development. Hyperglycemia in the mother as a consequence of excessive insulin resistance and insufficient insulin production in GDM or undiagnosed type 2/type 1 diabetes results in increased placental transfer of glucose to the fetus. The prolonged exposure of fetal pancreas to high levels of glucose leads to hyperinsulinemia characterized by increased lipogenesis and fat storage in a sex dependent manner [186]. Short-term offspring consequences of GDM such as macrosomia and neonatal hypoglycemia are the result of excess supply of glucose from maternal to fetal circulation. Further, in-utero exposure to the altered metabolic milieu in GDM can lead to long-term offspring consequences such as increased insulin resistance, adiposity and cardiovascular risk [60, 61, 71, 72, 187–189], by influencing the epigenetic programming during fetal development.

The first observation of the influence of the in-utero environment on fetal risk of developing chronic diseases was made by Barker et al. [190]. This study reported that babies who were small for gestational age with large placentas had circulatory adaptation in fetal stage which predisposes them to a high risk of developing CVD in adulthood [43, 190]. In addition, human population studies have shown that maternal nutrient status can program the pancreatic beta cell function in fetus and modify the risk of developing metabolic disorders in adult life [191, 192]. Epigenetic dysregulation is a remarkable link between neonatal exposure to uterine environment and later adult health. For example, in individuals who were prenatally exposed to the Dutch famine during World War II, it was identified 60 years later that they had distinct epigenetic changes in the form of decreased DNA methylation of the Insulin-like growth factor (IGF)2 gene compared to their same sex sibling who were unexposed to maternal undernutrition [193]. Further, studies in animal models which manipulated the maternal diet during pregnancy, and analyzed the epigenetic modifications in the fetus, provided insight into the developmental origins of metabolic diseases [7, 194, 195]. For example, changes in dietary methyl supplements fed to pregnant mice alters DNA methylation in the fetus and leads to associated phenotypic changes [7, 194], indicating that maternal nutritional status influences the long-term health of the fetus. There are numerous studies that report epigenetic changes in placenta and cord blood [15, 35, 36, 108, 196–198] in association with GDM. However, the impact of GDM on the epigenetic landscape of offspring metabolic tissues such as liver, pancreas, adipose tissue, and skeletal muscle is unknown as this is unlikely to be feasible except in patients where there is a clinical need to obtain biopsies. Overall, this suggests that the altered placental nutrient transport and intrauterine conditions associated with GDM affects the offspring’s epigenome, and susceptibility to disease later in life.

The field of EVs is expanding rapidly, and there is continuous exploration of the novel roles of these nanovesicles in various biological processes [144]. EVs carry a wide array of biological molecules as proteins, nucleic acids and lipids which mediates changes in the gene expression and phenotypic response in recipient cells [144, 146]. EVs can be key mediators of epigenetic signals by transferring non-coding RNAs including miRNAs and long non-coding RNAs, which can modify proteins involved in DNA methylation and histone modifications [157, 158, 161, 163–165, 167–169, 199]. EVs are reported to be involved in mediating key biological processes which underlie the metabolic adaptations in healthy pregnancies, and those complicated by GDM [17, 26, 28–31]. The most studied EVs in pregnancy are the total circulating EVs, which originate from different maternal tissues [19, 26, 27, 30]. In addition, EVs derived from the placenta form a critical link between the maternal and fetal system and are hence studied for their involvement in physiological and pathological processes [19, 28, 29]. However, there is a lack of studies on EVs of fetal origin due to an inability to obtain fetal EVs, and hence there is a gap in the understanding of their effects on target tissues. Since EVs can act as mediators of epigenetic signals, the EVs originating from fetal tissues can potentially propagate the metabolic signals between fetal organs and mediate the epigenetic programming in response to altered in-utero metabolic exposure in GDM.

**Fig. 2:**
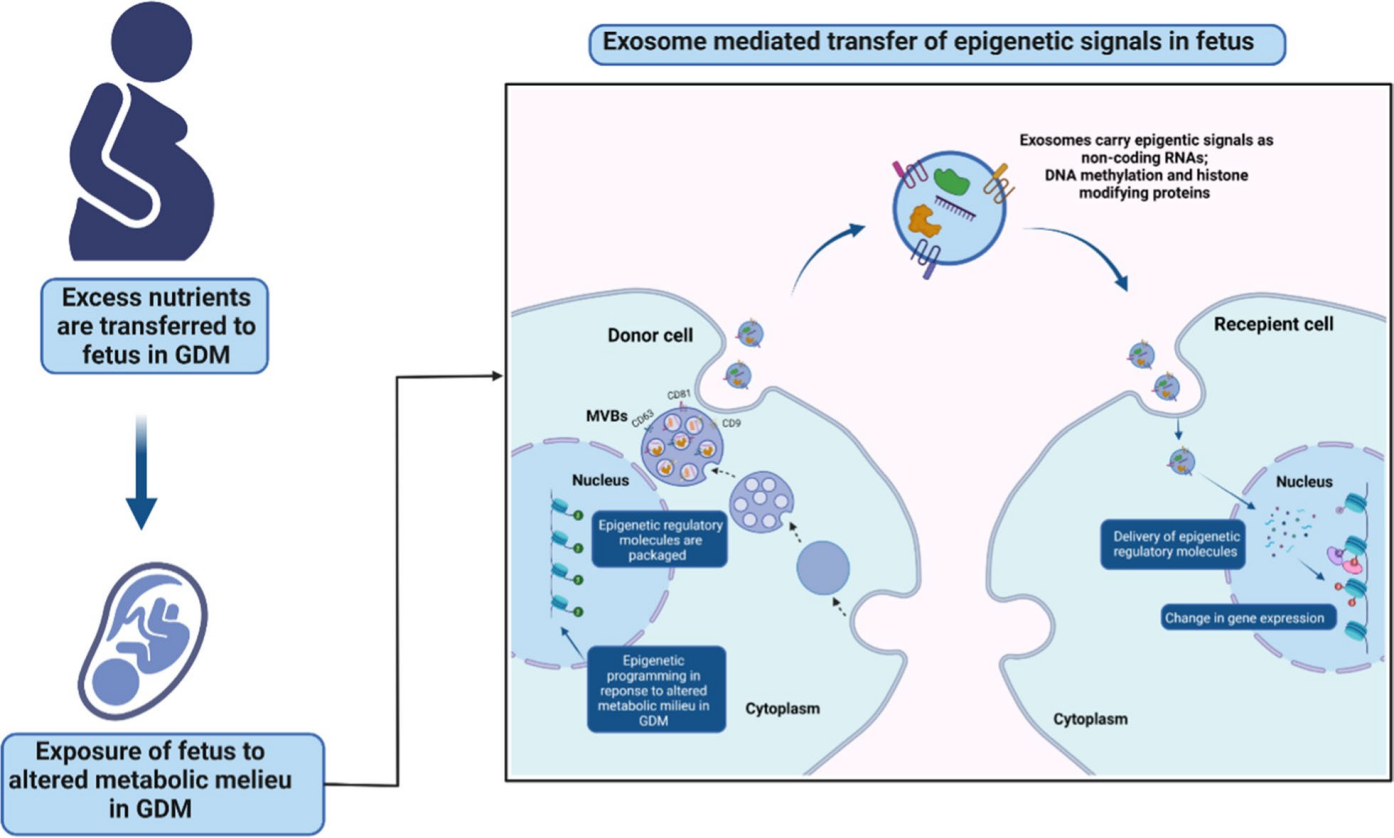
Schematic illustration of the potential role of EVs in mediating epigenetic signals in fetal metabolic reprogramming in GDM. In GDM, excess nutrients are supplied to the fetus via placental transfer which leads to exposure of fetal cells to the altered metabolic milieu. The fetal cells respond to the altered uterine microenvironment by epigenetic alterations in metabolic genes. EVs can potentially mediate the transfer of epigenetic signals between fetal cells thus mediating the change in the epigenetic landscape, and susceptibility to future diseases

Another critical question is whether the maternal EVs can cross the placental barriers and transfer epigenetic signals to fetal cells. Although, placental EVs have been reported in the cord blood [19], the mechanism by which EVs from the maternal side of the placenta can cross the placental barrier to reach fetal blood is not clearly understood. Future studies in in-vitro settings and animal models should investigate whether- and how-the transfer of epigenetic modifying cargo will affect the susceptibility to future diseases, including CVD in offspring born to GDM mothers. Figure 2 shows EV-mediated transfer of epigenetic signals which can potentially lead to epigenetic reprogramming in recipient cells in a fetus exposed to GDM.

## Conclusion and future directions

The development of metabolic disorders in life is dependent on genetic and epigenetic factors. The genetic factors are those that cause a change in the individual’s DNA sequence, whereas the epigenetic factors involve genetic control by factors other than alterations in DNA sequences. The effects of the external environment on genes and development of diseases involves epigenetic programming of the genome in response to the external cues. During pregnancy the changes in the maternal metabolism due to presence of metabolic disorders such as obesity, type 2 diabetes and GDM can have a crucial long-term impact on mothers and offspring. Available epidemiological data shows that the maternal intrauterine glycemic environment can epigenetically reprogram the fetus for the development of metabolic diseases in the future, including an increased risk of CVDs. Growing evidence reports the involvement of EVs of maternal and fetal origin as key players in the development of GDM, as well as the maternal and fetal pathology associated with GDM. Changes in the concentration, cargo, and bioactivity of total circulating and placental EVs have been described in GDM. However, there is no clear evidence showing direct involvement of EV signaling in the fetal programming for metabolic disease.

**Fig. 3:**
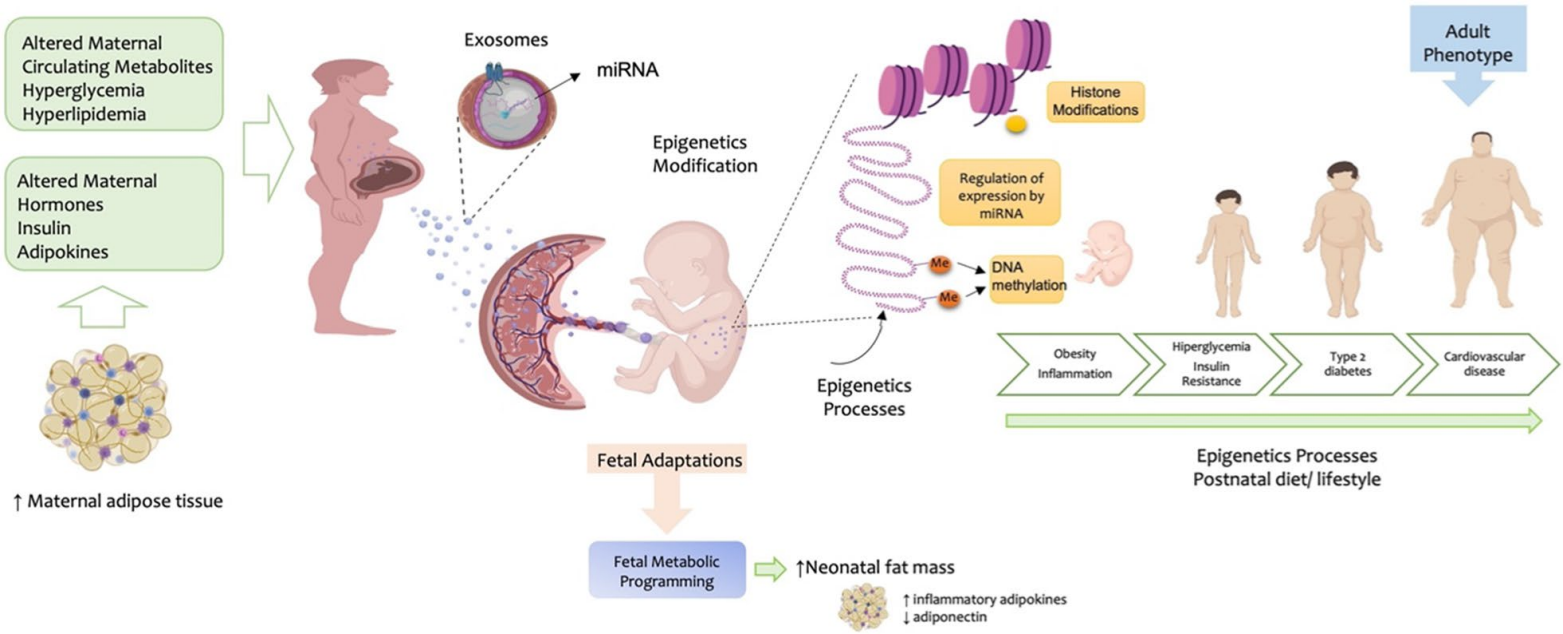
Maternal factors during pregnancy modify cell signals via EVs, which contribute to fetal metabolic programming, thus leaving a transgenerational imprint that affects health during adult life

In general, EVs are reported to be key players in the pathology of metabolic disorders. EV-mediated transfer of molecules between cells is reported to play a critical role in mediating the metabolic, inflammatory and vascular events associated with the development and progression of CVD. Most importantly, emerging studies show that changes in the external environment lead to changes in the molecular cargo of EVs, and the altered molecular cargo, when transferred to recipient cells, could epigenetically program these cells to have altered metabolic phenotypes. The transfer of epigenetic signals between cells via EVs have been demonstrated in the context of CVD, and associated risk factors such as type 2 diabetes and obesity. Hence, understanding the effect of EV signaling during pregnancy, on fetal programming for the development of metabolic diseases such as CVD, is a fertile area for future investigation. Knowledge regarding the factors that contribute to fetal programming is imperative for the development of interventions to stop or slow down the development and progression of CVD and associated pathologies (Fig. 3).

## Data Availability

Not applicable.
